# Meta-analysis of SNP-environment interaction with heterogeneity for overlapping data

**DOI:** 10.1038/s41598-021-82336-8

**Published:** 2021-01-28

**Authors:** Qinqin Jin, Gang Shi

**Affiliations:** 1 State Key Laboratory of Integrated Services Networks, Xidian University, 2 South Taibai Road, Xi’an, 710071 Shaanxi China; 2 Applied Science College, Taiyuan University of Science and Technology, Taiyuan, 030024 Shanxi China

**Keywords:** Genetics, Biomarkers, Diseases, Health care, Molecular medicine

## Abstract

Meta-analysis is a popular method used in genome-wide association studies, by which the results of multiple studies are combined to identify associations. This process generates heterogeneity. Recently, we proposed a random effect model meta-regression method (MR) to study the effect of single nucleotide polymorphism (SNP)-environment interactions. This method takes heterogeneity into account and produces high power. We also proposed a fixed effect model overlapping MR in which the overlapping data is taken into account. In the present study, a random effect model overlapping MR that simultaneously considers heterogeneity and overlapping data is proposed. This method is based on the random effect model MR and the fixed effect model overlapping MR. A new way of solving the logarithm of the determinant of covariance matrices in likelihood functions is also provided. Tests for the likelihood ratio statistic of the SNP-environment interaction effect and the SNP and SNP-environment joint effects are given. In our simulations, null distributions and type I error rates were proposed to verify the suitability of our method, and powers were applied to evaluate the superiority of our method. Our findings indicate that this method is effective in cases of overlapping data with a high heterogeneity.

## Introduction

Genome-wide association studies (GWASs) are effective for the identification of single nucleotide polymorphisms (SNPs) associated with complex traits or disease^1–3^. Meta-analysis^4–8^, which combines the results of multiple studies, is a common method used to increase the sample size^5,7,9^, which can reduce false positive results, increase power, and increase the probability of finding new associations. The fixed effect model is commonly used for meta-analysis, in which the effects between studies are assumed to be equal. However, in recent studies, meta-analyses have been employed using new designs, by combining different related traits or diseases^10,11^, environments^12^, populations^13^, tissues^14^, and cancer types^15,16^. These combinations lead to different effect sizes between studies, which is called heterogeneity^17^. Thus, the fixed effect model is not suitable. The traditional random effect model^4^ take heterogeneity into account ,but implicitly assumes a conservative null hypothesis model. Therefore, it provides a power even lower than the fixed model. A modified random effect model method^18^ was proposed to overcome this problem and be widely used in various analyses^12,14,17,19,20^.

However, in practice, there are many overlapping individuals between studies. This may be caused unintentionally or intentionally by the researchers. If these overlapping individuals exist but they are ignored, spurious associations may occur^21,22^. In recent years, researchers have proposed several methods for overlapping data^16,17,21–25^. These methods are all used to test the main effects of SNPs. Lin^21^ proposed a correlation matrix and applied it to the fixed model method for overlapping data among studies. Han^22^ transformed the covariance structure of data, which then became a form of diagonal matrix. This transformation makes Lin’s method more flexible, which can be widely applied by meta-analysis methods, such as the random effects model. Based on the modified random effect model method^18^, Lee^17^ proposed a new method for overlapping data. This method combined the fixed effect model and random effect model, which gave a higher power regardless of the heterogeneity.

The meta-regression method (MR)^26^ is a powerful and robust method in a fixed-effect model. This method has two steps. First, individuals in each study are divided into several groups based on the distribution of environmental variables, where the number of individuals in each group is equal. In each group, linear regression is used to estimate the main effects, standard errors, and mean environment variables of the SNP. Second, researcher collects all the above results and performs a meta-regression to investigate interactions between SNP and the environment. It can be used to test for the main effects of SNP, SNP-environment interactions, and joint effects. In addition, this method is considered to be a robust method when confounding effects exist, such as interactions between covariates and genetic effects or interactions between covariates and environmental factors^27^. Based on Lin’s method^21^ and Han’s method^22^, we extended MR to overlapping MR (OMR)^28^. This method is designed for SNP-environment interactions under the fixed effect model, as well as for overlapping data. We also extended MR to account for heterogeneity; that is, we added random effects of the SNP and SNP-environment interactions to the fixed-effect SNP-environment interaction model. This method is denoted as the random effect MR (RMR)^29^, which gives a higher power than MR under the fixed-effect model when heterogeneity exists. The Q-Q plot of the null distribution obtained by MR will shift upward when overlapping data exists. The more overlapping the data, the more obvious the deviation will be. The fixed effect model OMR^28^ controls spurious associations caused by overlapping data. When heterogeneity exists and is large, the power of RMR is higher than that of MR. Similarly, when overlapping data and heterogeneity exist, the power obtained by OMR will also be affected by heterogeneity, and the power it provides will be reduced. However, no study has yet considered this condition.

In this paper, inspired by OMR and Lee’s method^17^ which is proposed for testing SNP main effect with overlapping data, we propose random effect overlapping MR (ROMR) which is a new method to consider overlapping data based on the RMR ^29^. Our method is designed to test the SNP-environment interaction effect or the SNP and SNP-environment joint effects with overlapping data. This paper is organized as follows. In the Materials and Methods section, we introduce the correlation matrix into the RMR. We also present a new method to calculate the likelihood function. In the Results section, we carry out simulations to examine the null distribution, type I error rate, and power of our method. We also compare our method with the OMR. In the Discussion and Conclusion section, the results of this paper are analyzed and used to draw conclusions.

## Materials and methods

### Fixed effect overlapping MR

OMR is a method that extends from fixed effect MR, which is a powerful and robust method under the condition of independent data. This method has two procedures. First, by continuous or dichotomous environmental exposure distribution, each study is divided into several groups. In this process, each group is a subset of the study, and percentiles of the environmental exposure can be used to divide the study into several groups with approximately the same sample sizes. Then, in each group, the coefficient and variance of the main effects of SNPs are estimated. Second, the main effect of SNP and its corresponding standard deviation in each group are collected for regression analysis. Then, either the overall mean SNP-environment effect and its variance is estimated, or the mean SNP and SNP-environment joint effect vector and its variance matrix are estimated.

Assume that the environmental exposure is continuous and ${\widehat{\beta }}_{ij}$ is set to be the estimation of the main effects of SNPs in the *i*-th study and *j*-th group, where subscript $i=\mathrm{1,2},\dots ,n$ is the sample size of studies and subscript $j=\mathrm{1,2},\dots , {n}_{i}$ is the sample size of the groups in the *i*-th study. ${\widehat{e}}_{ij}$ and ${E}_{ij}$ are the standard error and mean environment exposure of the *i*-th study and *j*-th group, respectively. Under the second OMR procedure, the formula for the environment-dependent SNP effect $\widehat{{\varvec{\beta}}}$ can be expressed in the following form:

$$\widehat{{\varvec{\beta}}}={\varvec{X}}{\varvec{\upalpha}}+{\varvec{\varepsilon}}$$

where

$$\begin{aligned} \hat{\user2{\beta }} & = \left( {\begin{array}{*{20}c} {\hat{\user2{\beta }}_{1} } \\ {\begin{array}{*{20}c} {\hat{\user2{\beta }}_{2} } \\ \vdots \\ \end{array} } \\ {\hat{\user2{\beta }}_{{\varvec{n}}} } \\ \end{array} } \right),\hat{\user2{\beta }}_{{\varvec{i}}} = \left( {\begin{array}{*{20}c} {\hat{\beta }_{i1} } \\ {\begin{array}{*{20}c} {\hat{\beta }_{i2} } \\ \vdots \\ \end{array} } \\ {\hat{\beta }_{{in_{i} }} } \\ \end{array} } \right),{\varvec{X}} = \left( {\begin{array}{*{20}c} {{\varvec{X}}_{1} } \\ {\begin{array}{*{20}c} {{\varvec{X}}_{2} } \\ \vdots \\ \end{array} } \\ {{\varvec{X}}_{{\varvec{n}}} } \\ \end{array} } \right),{\varvec{X}}_{{\varvec{i}}} = \left( {\begin{array}{*{20}c} 1 & {E_{i1} } \\ {\begin{array}{*{20}c} 1 \\ \vdots \\ \end{array} } & {\begin{array}{*{20}c} {E_{i2} } \\ \vdots \\ \end{array} } \\ 1 & {E_{{in_{i} }} } \\ \end{array} } \right), \\ {\varvec{\varepsilon}} & = \left( {\begin{array}{*{20}c} {{\varvec{\varepsilon}}_{1} } \\ {\begin{array}{*{20}c} {{\varvec{\varepsilon}}_{2} } \\ \vdots \\ \end{array} } \\ {{\varvec{\varepsilon}}_{{\varvec{n}}} } \\ \end{array} } \right),{\varvec{\varepsilon}}_{{\varvec{i}}} = \left( {\begin{array}{*{20}c} {\varepsilon_{i1} } \\ {\begin{array}{*{20}c} {\varepsilon_{i2} } \\ \vdots \\ \end{array} } \\ {\varepsilon_{{in_{i} }} } \\ \end{array} } \right) \\ {\varvec{\alpha}} & = \left( {\begin{array}{*{20}c} {\alpha_{0} } \\ {\alpha_{1} } \\ \end{array} } \right), {\varvec{\varSigma}}= \left( {\begin{array}{*{20}c} {{\varvec{\varSigma}}_{1} } & \cdots & 0 \\ \vdots & \ddots & \vdots \\ 0 & \cdots & {{{\varvec{\Sigma}}}_{{\text{n}}} } \\ \end{array} } \right),{\varvec{\varSigma}}_{i} = \left( {\begin{array}{*{20}c} {\hat{e}_{i1} } & \cdots & 0 \\ \vdots & \ddots & \vdots \\ 0 & \cdots & {\hat{e}_{{in_{i} }} } \\ \end{array} } \right) \\ \end{aligned}$$

and $${\varepsilon }_{ij}\sim N(0,{\widehat{e}}_{ij})$$ $$i=\mathrm{1,2},\dots ,n, j=\mathrm{1,2},\dots , {n}_{i}$$*.*

Let ${\varvec{C}}$ be the correlation matrix. The element of this matrix is

$${\gamma }_{{i}_{h}{j}_{k}}\approx {n}_{{i}_{h}{j}_{k}}/\sqrt{{n}_{{i}_{h}}{n}_{{j}_{k}}}$$

where the subscript ${n}_{{i}_{h}}$ and ${n}_{{j}_{k}}$ are presented as the size of the $h$-th group of study $i$ and $k$-th group of study *j*, respectively, and ${n}_{{i}_{h}{j}_{k}}$ is the size of the overlap individuals between the $h$-th and $k$-th group.

The covariance matrix of this method is

$$\boldsymbol{\Omega }={{\varvec{\Sigma}}}^{1/2}{\varvec{C}}{\boldsymbol{\Sigma }}^{1/2}$$

It has another form as

$$\boldsymbol{\Omega }={{\varvec{d}}{\varvec{i}}{\varvec{a}}{\varvec{g}}({{\varvec{e}}}^{\mathbf{^{\prime}}}{({\boldsymbol{\Sigma }}^{1/2}{\varvec{C}}{\boldsymbol{\Sigma }}^{1/2})}^{-1})}^{-1}$$

where ${\varvec{e}}=\left(\mathrm{1,1},\dots ,1\right)$ and its length is the sum of all group sizes.

The formula of the linear unbiased estimators $\widehat{\boldsymbol{\alpha }}$ and $\mathrm{Cov}\left(\widehat{\boldsymbol{\alpha }}\right)$ are expressed as follows

$$\widehat{\boldsymbol{\alpha }}={\left({{\varvec{X}}}^{\mathrm{^{\prime}}}{\boldsymbol{\Omega }}^{-1}{\varvec{X}}\right)}^{-1}{{\varvec{X}}}^{\mathrm{^{\prime}}}{\boldsymbol{\Omega }}^{-1}\widehat{{\varvec{\beta}}}$$

$${\widehat{\alpha }}_{2}=\left(\mathrm{0,1}\right)\widehat{\boldsymbol{\alpha }}$$

$$\mathrm{Cov}\left(\widehat{\boldsymbol{\alpha }}\right)={\left({{\varvec{X}}}^{\mathrm{^{\prime}}}{\boldsymbol{\Omega }}^{-1}{\varvec{X}}\right)}^{-1}$$

$${\mathrm{Cov}\left(\widehat{\boldsymbol{\alpha }}\right)}_{22}=(\mathrm{0,1})\mathrm{Cov}\left(\widehat{\boldsymbol{\alpha }}\right)\left(\begin{array}{c}0\\ 1\end{array}\right)$$

Under null distribution ${\widehat{\alpha }}_{2}=0$ and $\widehat{\boldsymbol{\alpha }}=0$, the Wald statistic of the SNP-environment interaction effect and the SNP and SNP-environment joint effects follow 1 and 2 degrees of freedom (df) ${\upchi }^{2}$ distribution, respectively.

### Random effect overlapping MR

This method is an extension of the OMR^28^ and the recently proposed RMR^29^. Under this method, the random effects for the SNP main and SNP-environment interaction are denoted as $\gamma$. The environment-dependent SNP effect $\widehat{{\varvec{\beta}}}$ is presented as follows:

$$\widehat{{\varvec{\beta}}}=\boldsymbol{ }{\varvec{X}}{\varvec{\upalpha}}+{\varvec{Z}}{\varvec{\gamma}}+{\varvec{\varepsilon}}$$

where

$${\varvec{Z}}=\left(\begin{array}{ccc}{{\varvec{Z}}}_{1}& \cdots & 0\\ \vdots & \ddots & \vdots \\ 0& \cdots & {{\varvec{Z}}}_{n}\end{array}\right),\boldsymbol{ }{\varvec{\gamma}}=\left(\begin{array}{c}{{\varvec{\gamma}}}_{1}\\ \begin{array}{c}{{\varvec{\gamma}}}_{2}\\ \vdots \end{array}\\ {{\varvec{\gamma}}}_{n}\end{array}\right)$$

and

$${{\varvec{\gamma}}}_{i}=\left(\begin{array}{c}{\gamma }_{i0}\\ {\gamma }_{i1}\end{array}\right)$$

Here, variable ${\gamma }_{i0}$ is denoted as the random main effect of SNP in the *i*-th study and ${\gamma }_{i1}$ is denoted as the random effect of SNP-environment interaction in the *i*-th study. The vector $${{\varvec{\gamma}}}_{i}=\left(\begin{array}{c}{\gamma }_{i0}\\ {\gamma }_{i1}\end{array}\right)$$ follows a bivariate normal distribution with $$\left(\begin{array}{c}{\gamma }_{0i}\\ {\gamma }_{1i}\end{array}\right)\sim \mathrm{N}(0,{{\varvec{D}}}_{\mathrm{s}})$$. The variable $\widehat{{\varvec{\beta}}}$ followed a multivariate normal distribution, as follows:

$$\widehat{{\varvec{\beta}}}\sim \mathrm{N}\left({\varvec{X}}{\varvec{\upalpha}},{\varvec{V}}\right)$$

where in the overlapping condition ${\varvec{V}}={\varvec{Z}}{\varvec{D}}{{\varvec{Z}}}^{\boldsymbol{^{\prime}}}+{\boldsymbol{\Sigma }}^{1/2}{\varvec{C}}{\boldsymbol{\Sigma }}^{1/2}$ is a real symmetric matrix**,** denote ${\lambda }_{1}, {\lambda }_{2},\dots ,{\lambda }_{M}$where $M=\sum_{i=1}^{n}{n}_{i}$ as the eigenvalues of matrix ${\varvec{V}}$, denote ${\xi }_{1}, {\xi }_{2},\dots ,{\xi }_{M}$ as the orthogonal eigenvector of matrix ${\varvec{V}}$, that is to say, $\left|\left({\xi }_{1}, {\xi }_{2},\dots ,{\xi }_{M}\right)\right|=1$ and $\left({\xi }_{1}, {\xi }_{2},\dots ,{\xi }_{M}\right)={\left({\xi }_{1}, {\xi }_{2},\dots ,{\xi }_{M}\right)}^{-1}$. Then $\left|{\varvec{V}}\right|={\lambda }_{1}*{\lambda }_{2}*\dots *{\lambda }_{M}$.

The likelihood function under this model can be written as

$${l}_{1}={\sum }_{i=1}^{M}\mathrm{ln}\left|{\lambda }_{i}^{1}\right|+{\left(\widehat{{\varvec{\beta}}}-{\varvec{X}}{\varvec{\upalpha}}\right)}^{^{\prime}}{V}^{-1}\left(\widehat{{\varvec{\beta}}}-{\varvec{X}}{\varvec{\upalpha}}\right)+\sum_{i=1}^{n}{n}_{i}\mathrm{ln}\left(2\pi \right)$$

The estimation of this likelihood function is given by the minimum variance quadratic unbiased estimator (MIVQUE(0))^30,31^ and Newton–Raphson algorithms^32–34^. The detailed process is given in Jin^29^.

### Test of SNP-environment interaction

Under this test, we suppose that there is no interaction effect and no interaction heterogeneity, that is, ${\alpha }_{1}=0$ and $${{\varvec{D}}}_{\mathrm{s}}=\left(\begin{array}{cc}{u}_{1}^{2}& 0\\ 0& 0\end{array}\right)$$. The reduced model can then be given as follows:

$$\widehat{{\varvec{\beta}}}=\boldsymbol{ }{\varvec{X}}{\alpha }_{0}+{\varvec{Z}}{\gamma }_{0}+{\varvec{\varepsilon}}$$

where

$${\varvec{X}}={\varvec{Z}}=\left(\begin{array}{c}{{\varvec{X}}}_{1}\\ \begin{array}{c}{{\varvec{X}}}_{2}\\ \vdots \end{array}\\ {{\varvec{X}}}_{n}\end{array}\right),{{\varvec{X}}}_{i}={\left(\mathrm{1,1},\cdots ,1\right)}^{^{\prime}}$$

and the dimension of ${{\varvec{X}}}_{i}$ is ${n}_{i}$. In this model, $\widehat{{\varvec{\beta}}}\sim \mathrm{N}\left({\varvec{X}}{\alpha }_{0},{\varvec{V}}\right)$, the covariance matrix is ${\varvec{V}}={\varvec{Z}}{\varvec{D}}{{\varvec{Z}}}^{\boldsymbol{^{\prime}}}+{\boldsymbol{\Sigma }}^{1/2}{\varvec{C}}{\boldsymbol{\Sigma }}^{1/2}$**.** Denote ${\lambda }_{1}^{1}, {\lambda }_{2}^{1},\dots ,{\lambda }_{M}^{1}$ where $M=\sum_{i=1}^{n}{n}_{i}$ as the eigenvalues of matrix ${\varvec{V}}$, then $\left|{\varvec{V}}\right|={\lambda }_{1}^{1}*{\lambda }_{2}^{1}*\dots *{\lambda }_{M}^{1}$*.*

The -2 times of the log likelihood for this model is

$${l}_{2}={\sum }_{i=1}^{M}\mathrm{ln}\left|{\lambda }_{i}^{1}\right|+{\left(\widehat{{\varvec{\beta}}}-{\varvec{X}}{\alpha }_{0}\right)}^{^{\prime}}{V}^{-1}\left(\widehat{{\varvec{\beta}}}-{\varvec{X}}{\alpha }_{0}\right)+{\sum }_{i=1}^{n}{n}_{i}\mathrm{ln}(2\pi )$$

As in Jin^29^, the likelihood ratio statistic for the test of SNP-environment interaction is given as follows:

$${\mathrm{L}}_{\mathrm{I}}={\widehat{l}}_{2}-{\widehat{l}}_{1}$$

where ${\widehat{l}}_{1}$ is the minimum of ${l}_{1}$ and ${\widehat{l}}_{2}$ is the minimum of ${l}_{2}$. The statistic ${\mathrm{L}}_{\mathrm{I}}$ asymptotically follows an equal mixture of 2 df ${\upchi }^{2}$ distribution and 3 df ${\upchi }^{2}$ distribution. Its p-value is calculated by 0.5 (P (${\upchi }_{2}^{2}>{\mathrm{L}}_{\mathrm{I}}$) + P(${\upchi }_{3}^{2}>{\mathrm{L}}_{\mathrm{I}}$))^29^.

### Joint test of SNP and SNP-environment

Under this test, we suppose that $\boldsymbol{\alpha }=0$ and $${{\varvec{D}}}_{\mathrm{s}}=\left(\begin{array}{cc}0& 0\\ 0& 0\end{array}\right)$$, that is to say, no SNP main, SNP-environment interaction fixed effects, and no corresponding heterogeneity. The null model can be given as follows:

$$\widehat{{\varvec{\beta}}}={\varvec{\varepsilon}}$$

Then, $\widehat{{\varvec{\beta}}}\sim \mathrm{N}\left(0,{\varvec{V}}\right)$ and ${\varvec{V}}={{\varvec{\Sigma}}}^{1/2}\mathbf{C}{{\varvec{\Sigma}}}^{1/2}$**.** The eigenvalues of the covariance matrix ${\varvec{V}}$ are denoted as ${\uplambda }_{1}^{0},{\uplambda }_{2}^{0},\dots ,{\uplambda }_{M}^{0}$, where $M=\sum_{i=1}^{n}{n}_{i}$. Then $\left|{\varvec{V}}\right|={\lambda }_{1}^{0}*{\lambda }_{2}^{0}*\dots *{\lambda }_{M}^{0}$.

The -2 times of the log likelihood for this model is

$${l}_{0}={\sum }_{i=1}^{M}\mathrm{ln}\left|{\lambda }_{i}^{0}\right|+{\left(\widehat{{\varvec{\beta}}}-{\varvec{X}}{\mathrm{\alpha }}_{0}\right)}^{^{\prime}}{{\varvec{V}}}^{-1}\left(\widehat{{\varvec{\beta}}}-{\varvec{X}}{\mathrm{\alpha }}_{0}\right)+{\sum }_{i=1}^{n}{n}_{i}\mathrm{ln}(2\pi )$$

The likelihood ratio statistic for the joint test of the SNP main and SNP-environment is given as follows:

$${\mathrm{L}}_{\mathrm{J}}={\widehat{l}}_{0}-{\widehat{l}}_{1}$$

where ${\widehat{l}}_{0}$ is the evaluated value of ${l}_{0}$. The statistic ${\mathrm{L}}_{\mathrm{J}}$ asymptotically follows a $\upxi$:0.5:(0.5-$\upxi$) mixture of 3 df ${\upchi }^{2}$ distribution, 4 df ${\upchi }^{2}$ distribution, and 5 df ${\upchi }^{2}$ distribution. The value of $\xi$ depends on the given data and is solved by the information matrix. The p-value is calculated by(0.5-$\upxi$) P (${\upchi }_{3}^{2}>{\mathrm{L}}_{\mathrm{J}}$) + 0.5P(${\upchi }_{4}^{2}>{\mathrm{L}}_{\mathrm{J}}$)+ $\upxi$ P (${\upchi }_{5}^{2}>{\mathrm{L}}_{\mathrm{J}}$)^29^.

### Ethics approval

The authors have no ethical conflicts to disclose.

## Results

### Simulation

The relationship among the quantitative trait $Y$, the genotype of SNP $G$, and the environmental variable $E$ is presented as follows:

$$Y=({\beta }_{G}+{\gamma }_{G})G+({\beta }_{G\times E}+{\gamma }_{G\times E})G\times E+{\beta }_{E}E+\varepsilon$$

The quantitative trait $Y$ was simulated as a standardized normal distribution, that is, with a mean of 0 and a variance of 1. SNP was assumed as an additive genetic effect, and its minor allele frequency was 0.3. $G$ was coded as the number of minor alleles. In each study, 1000 points following a standard uniform distribution were generated. If these points fell in $[0,{0.3}^{2}]$, then $G$ was set to 2. If these points fell in $[{0.3}^{2},{0.3}^{2}+2\cdot 0.3\cdot (1-0.3)]$, then $G$ was set to 1, else $G$ was set to 0. The environmental variable $E$ was also simulated as a standardized normal distribution. A 10% variation in $Y$ was explained by the environmental term ${\beta }_{E}E$. Fixed effects ${\beta }_{G}$, ${\beta }_{G\times E}$ and random effects ${\gamma }_{G}$, ${\gamma }_{G\times E}$ changed in simulation datasets. The random error $\varepsilon$ was normally distributed, with a zero mean and a variance chosen so that the variance of *Y* was 1. In our simulation, we generated 1000 replications, each replication had 12 studies, each study had 1000 unrelated individuals, and each individual had one quantitative trait $Y$, one environmental variable $E$, and one SNP $G$. Across all the studies, 100 and 400 overlapping individuals were observed. Before analysis, the individuals in each study were divided into five groups according to the distribution of $E$. The main effects of SNP and standard errors were estimated by linear regression. The mean of the environmental variables $E$ of each stratum was then calculated.

To test the null distribution of statistics for the SNP-environment interaction, we assumed that both the SNP-environment interaction and its corresponding heterogeneity were zero. That is to say, ${\beta }_{G\times E}=0$ and ${\gamma }_{G\times E}=0$. The main effect of SNP ${\beta }_{G}$ was set to a square root of 0.1, and the random effect of this effect was set to be normally distributed, with a mean of 0 and a variance of 0.02. We calculated the minimum estimates of the likelihood functions ${l}_{1}$ and ${l}_{2}$, such that the statistic ${\mathrm{L}}_{\mathrm{I}}$ could be obtained. Finally, empirical P-values were calculated using a 0.5:0.5 mixture of 2 and 3 df ${\upchi }^{2}$ distributions, which was the theoretical distribution of the interaction test. Then, these were compared with expected values following a uniform distribution between 0 and 1. A Q–Q plot was drawn through the two types of P-values. To test the null distribution of statistics for the SNP and SNP-environment joint effects, we assumed that all the fixed effects and random effects of the SNP and SNP-environment interaction were zero. That is, ${\beta }_{G}$=${\beta }_{G\times E}$=${\gamma }_{G}$=${\gamma }_{G\times E}$=0.We calculated the likelihood ratio statistic ${\mathrm{L}}_{\mathrm{J}}$ by estimating the minimum of the likelihood function ${\widehat{l}}_{0}$ and ${\widehat{l}}_{1}$. The empirical P-values were calculated as a $\upxi$:0.5:(0.5-$\upxi$) mixture of 3 df ${\upchi }^{2}$ distribution, 4 df ${\upchi }^{2}$ distribution, and 5 df ${\upchi }^{2}$ distribution. The value of $\xi$ was data-dependent and calculated using Fisher information.

To test the powers of the SNP-environment interaction effect, we set the fixed effects of the SNP main ${\beta }_{G}$ and SNP-environment interaction ${\beta }_{G\times E}$ to $\sqrt{0.002}$. The random effect of SNP main ${\gamma }_{G}$ was normally distributed, followed $E\sim \mathrm{N}(\mathrm{0,0.015})$, variance of random effect of SNP-environment interaction ranging from 0.005 to 0.025, where each increased by 0.005. If the P-value of the test is less than 0.05, it was considered statistically significant. Experiments were repeated 1000 times, and the proportion of statistical significance was called empirical power. The OMR was also tested under this simulation. To test for the SNP and SNP-environment interaction joint effects, the fixed effects of ${\beta }_{G}$ and ${\beta }_{G\times E}$ were set to a square root of 0.002. The random effects of ${\gamma }_{G}$ and ${\gamma }_{G\times E}$ were set normally distributed with a mean of 0 and a variance ranging from 0.005 to 0.025.

### Null distribution

**Figure 1:**
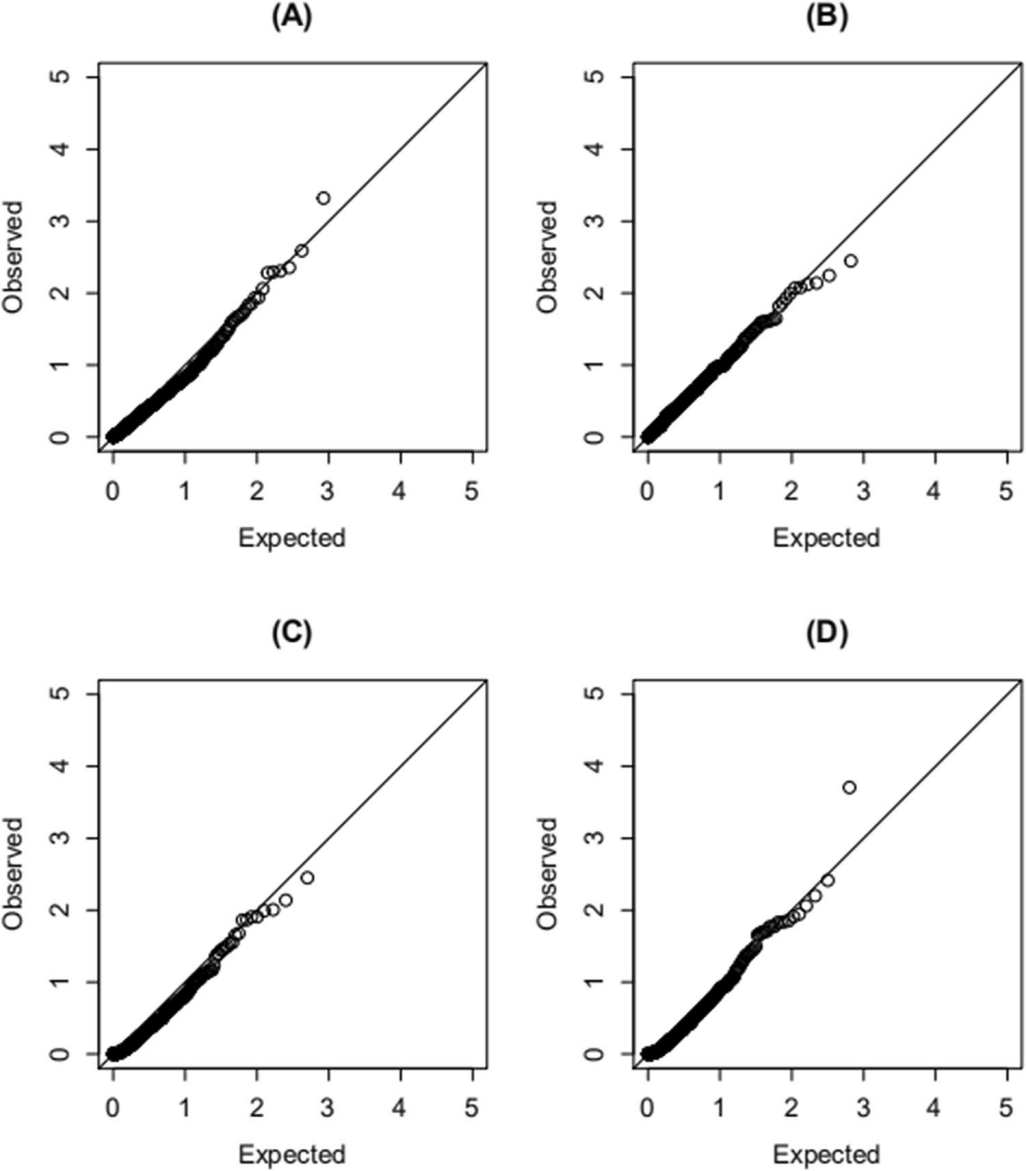
Q–Q plots of the null distributions in the test for SNP-environment interaction effects and the joint test for SNP and SNP-environment interaction effects. (**A**, **B**) The tests for SNP-environment interaction effects with 100 and 400 overlapping individuals between any two studies. (**C**, **D**) The joint tests for SNP and SNP-environment interaction effects with 100 and 400 overlapping individuals between any two studies. The vertical axis is the—log10 (observed P value) from data analyzed under the null hypothesis, and the horizontal axis is the—log10 (expected P value).

As shown in Fig. 1A,B, these points are nearly standing on the diagonal line with 100 and 400 overlapping individuals between studies. This verifies that the method presented provides suitable distributions. In Fig. 1C,D, the empirical P-values are close to the expected ones with 100 and 400 overlapping individuals between any two studies, demonstrating the suitability of our distributions.

### Type I error rate

To better illustrate the performance of our method, we considered three different scenarios. In scenario 1, two different sample sizes were considered: (1) a study with 1000 individuals and (2) a study with 2000 individuals. In scenario 2, two different significance levels were considered: (1) 0.01 and (2) 0.05. In scenario 3, two different main effects of SNP were considered: (1) a square root of 0.1 and (2) a square root of 0.2. Table 1 presents the values of the type I error rates in the different scenarios for the test of the SNP-environment interaction with 10% overlapping data. Table 2 presents the values of the type I error rates in the different scenarios for the test of the SNP-environment interaction with 40% overlapping data. Table 3 presents the values of the type I error rates in the different scenarios for the test of the SNP and SNP-environment joint effects with 10% overlapping data. Table 4 presents the values of the type I error rates in the different scenarios for the test of the SNP and SNP-environment joint effects with 40% overlapping data. For the 1000 replications, the 95% confidence intervals for the estimated type I error rates of nominal levels 0.05 and 0.01are (0.036, 0.064) and (0.002, 0.018) respectively. For the 2000 replications, the 95% confidence intervals for the estimated type I error rates of nominal levels 0.05 and 0.01are (0.040, 0.060) and (0.004, 0.016) respectively. From these tables, we can see that all of the estimated type I error rates are in the confidence intervals for interaction tests and joint tests, this indicates that our method is valid.

**Table 1:** The values of type I error rates at different scenarios for the test of SNP-environment interaction with 10% overlapping data.

Individuals of study	Significance level	_SNP main effect_	
		$\beta_{{\text{G}}} = \sqrt {0.1}$	$\beta_{{\text{G}}} = \sqrt {0.2}$
1000	0.01	0.008	0.0156
	0.05	0.038	0.054
2000	0.01	0.012	0.011
	0.05	0.048	0.046

**Table 2:** The values of type I error rates at different scenarios for the test of SNP-environment interaction with 40% overlapping data.

Individuals of study	Significance level	_SNP main effect_	
		$\beta_{{\text{G}}} = \sqrt {0.1}$	$\beta_{{\text{G}}} = \sqrt {0.2}$
1000	0.01	0.011	0.009
	0.05	0.048	0.056
2000	0.01	0.011	0.006
	0.05	0.047	0.040

**Table 3:** The values of type I error rates at different scenarios for the test of SNP and SNP-environment joint effects with 10% overlapping data.

Individuals of study	Significance level	
	0.01	0.05
1000	0.008	0.038
2000	0.010	0.040

**Table 4:** The values of type I error rates at different scenarios for the test of SNP and SNP-environment joint effects with 40% overlapping data.

Individuals of study	Significance level	
	0.01	0.05
1000	0.008	0.050
2000	0.011	0.047

### Statistical power

**Figure 2:**
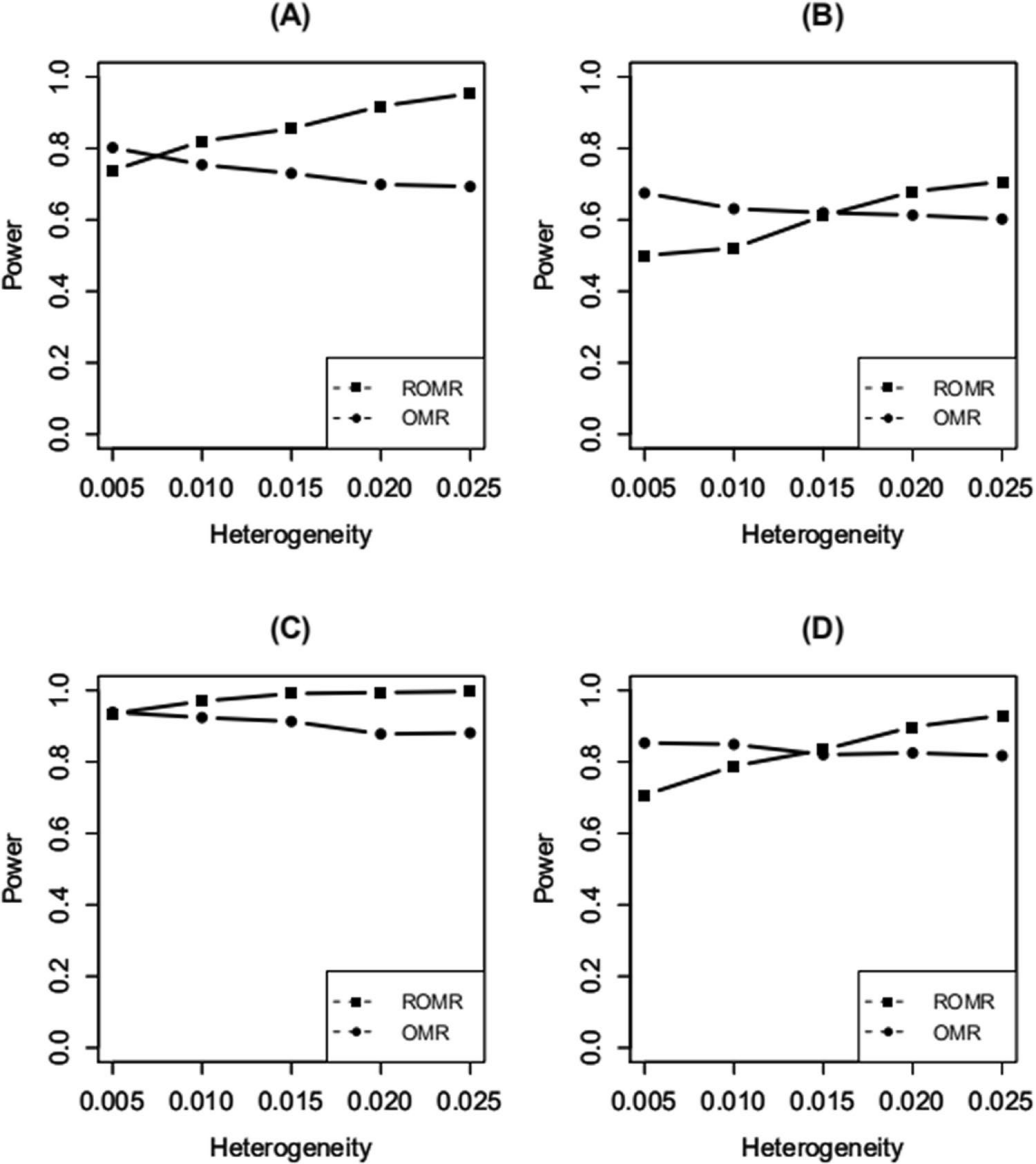
Statistical power of the test for SNP-environment interaction effects and the joint test for SNP and SNP-environment interaction effects. (**A**, **B**) The tests for SNP-environment interaction effects with 100 and 400 overlapping individuals between any two studies. (**C**, **D**) The joint tests for SNP and SNP-environment interaction effects with 100 and 400 overlapping individuals between any two studies. The vertical axis is the statistical power, and the horizontal axis is the heterogeneity effect.

The powers of ROMR and OMR are compared in Fig. 2A,B. The OMR gives higher powers when heterogeneity is low with 100 and 400 overlapping individuals between any two studies. The powers of this method decrease slightly with increase in heterogeneity, however, the powers of the ROMR increase rapidly with increase in heterogeneity. When the heterogeneity is high, the ROMR gives higher powers. This is due to the fact that when the heterogeneity is low, most of the statistical evidence of ${\text{L}}_{\text{I}}$ is obtained from the fixed effect of the interaction. In fact, the test statistics of ROMR are penalized by high degrees of freedom, yielding less power. When the heterogeneity is high, the OMR tested for the fixed effect only, the genetic effect tested by the ROMR is much larger than that of OMR. Thus, ROMR gives higher power^29^. As shown in Fig. 2C,D, although our method provides a similar tendency to interaction simulation, joint tests generally obtain higher results. This is because both the SNP and SNP-environment interaction are tested, thereby including more effects than the test for the interaction only.

**Figure 3:**
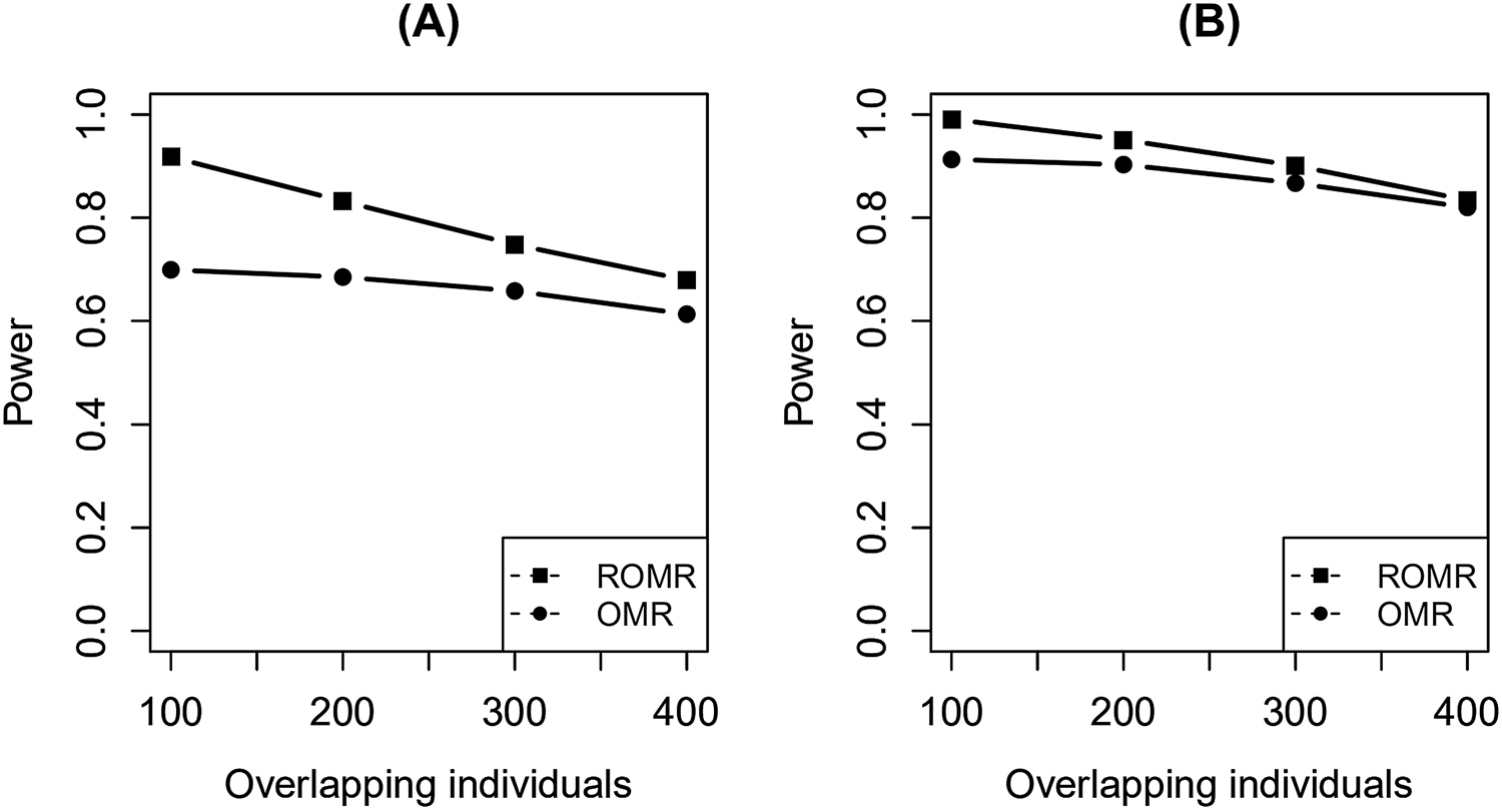
Statistical power of the test for SNP-environment interaction effects and the joint test for SNP and SNP-environment interaction effects with 100, 200, 300, 400 overlapping individuals under a fixed heterogeneity. (**A**) The tests for SNP-environment interaction effects with 100, 200, 300, 400 overlapping individuals under a fixed heterogeneity. (**B**) The joint tests for SNP and SNP-environment interaction effects with 100, 200, 300, 400 overlapping individuals under a fixed heterogeneity.

Table 5 and 6 present the powers under different levels of heterogeneity with different overlapping data. Table 5 shows the powers of the SNP-environment interaction, showing that the power decreased with an increase in the number of overlapping data. For ROMR, the greatest drop was 0.299; for OMR, the greatest drop was 0.127. However, in any case, when the heterogeneity was large, ROMR gave a higher power than OMR. Table 6 shows the powers of the SNP and SNP-environment interaction joint effects. Similar to the case of SNP-environment interaction, as the number of overlapping data increased, the power of ROMR was reduced faster than OMR. However, when the heterogeneity was large, ROMR gave a higher power than OMR. In order to more intuitively understand the impact of different overlapping data, Fig. 3 is given. We selected one set of parameters from Table 5 and 6. The variance of heterogeneity for SNP-environment interaction was fixed as 0.02 and the overlapping individuals were 100, 200, 300 and 400 as in the tables. As can be seen from Fig. 3A,B, with the increase of overlapping individuals, the powers of the two methods are decreasing gradually. The powers of SNP-environment interaction effects and the powers of the SNP and SNP-environment interaction joint effects have the same variation tendency.

**Table 5:** The powers under different heterogeneity with different overlapping data for the test of SNP-environment interaction.

Overlapping individuals	Methods	Heterogeneity				
		0.005	0.01	0.015	0.02	0.025
100	ROMR	0.738	0.820	0.855	0.918	0.953
	OMR	0.802	0.754	0.730	0.699	0.693
200	ROMR	0.622	0.701	0.797	0.832	0.890
	OMR	0.716	0.708	0.692	0.685	0.683
300	ROMR	0.519	0.617	0.670	0.748	0.793
	OMR	0.683	0.673	0.661	0.658	0.644
400	ROMR	0.500	0.521	0.611	0.679	0.707
	OMR	0.675	0.631	0.620	0.613	0.602

**Table 6:** The powers under different heterogeneity with different overlapping data for the test of SNP and SNP-environment joint effects.

Overlapping individuals	Methods	Heterogeneity				
		0.005	0.01	0.015	0.02	0.025
100	ROMR	0.935	0.970	0.991	0.994	0.997
	OMR	0.939	0.924	0.913	0.878	0.881
200	ROMR	0.873	0.921	0.951	0.979	0.989
	OMR	0.926	0.910	0.903	0.898	0.876
300	ROMR	0.784	0.865	0.901	0.952	0.957
	OMR	0.898	0.875	0.867	0.861	0.833
400	ROMR	0.707	0.788	0.834	0.898	0.930
	OMR	0.853	0.849	0.820	0.825	0.817

**Figure 4:**
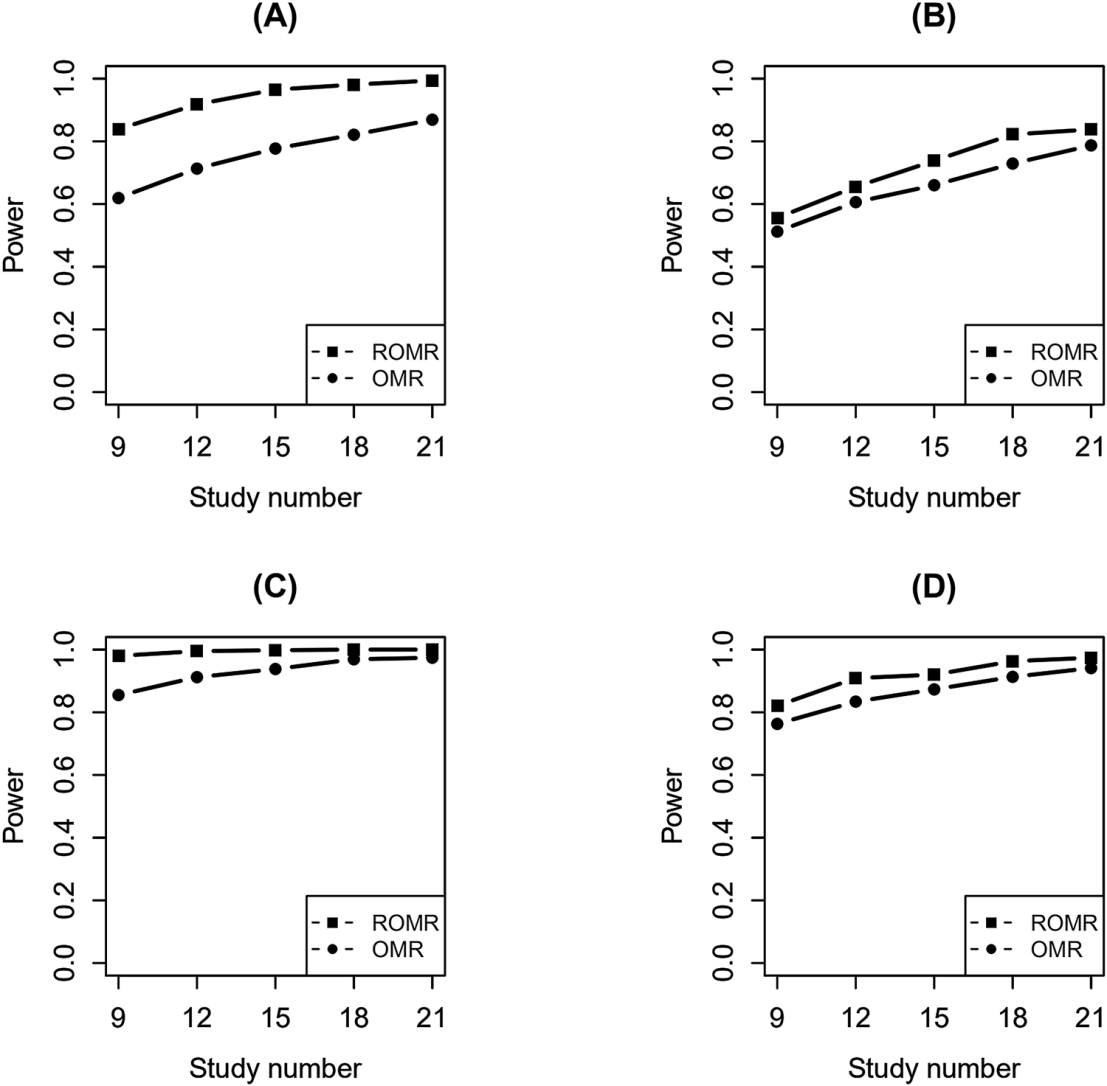
Statistical power of the test for SNP-environment interaction effects and the joint test for SNP and SNP-environment interaction effects with different number of studies (9, 12, 15, 18, 21) and with 100 and 400 overlapping individuals under a fixed heterogeneity. (**A**, **B**) The tests for SNP-environment interaction effects with different number of studies (9, 12, 15, 18, 21) and with 100 and 400 overlapping individuals under a fixed heterogeneity. (**C**, **D**) The joint tests for SNP and SNP-environment interaction effects with different number of studies (9, 12, 15, 18, 21) and with 100 and 400 overlapping individuals under a fixed heterogeneity.

Figure 4 present the powers under different number of studies. The variance of heterogeneity for SNP-environment was fixed as 0.02 and the numbers of studies were 9, 12, 15, 18, 21. Figure 4A,B present the powers of SNP-environment interaction effects under 100 and 400 overlapping individuals. Figure 4C,D present the powers of SNP and SNP-environment interaction joint effects under 100 and 400 overlapping individuals. As can be seen from these figures, with the increase of the numbers of studies, the powers of the two methods are increasing gradually.

## Discussion

In contrast to the calculation of the likelihood function performed by Lee^17^, we only changed the calculation of $\mathrm{ln}\left|V\right|$. The covariance matrix $V$ is a real symmetric matrix that can be diagonalized in a similar manner. That is to say, $V$ can be written in the form of ${P}^{-1}\mathrm{\Lambda P}$, where $P$ is the matrix of eigenvectors and $\Lambda$ is the vector of eigenvalues. Then, $\mathrm{ln}\left|V\right|=\sum \mathrm{ln}\left|{\lambda }_{i}\right|$, where ${\lambda }_{i}$ is the eigenvalue of the covariance matrix $V$. The other terms of the likelihood function are the same as in the random effect model MR. Thus, the computational complexity of our method is much less than that of Lee’s method. We can also compute $\mathrm{ln}\left|V\right|$ using OMR or by combining both of the above mentioned methods.

As in Lee^17^, our method can also be combined with OMR, providing a higher power, regardless of the level of heterogeneity. This method focuses on heterogeneity, it designs statistic as follows

$$\mathrm{L}=\left\{\begin{array}{l}{\mathrm{L}}_{\mathrm{R}}\quad if\quad {p}_{R}\le {p}_{F} \\ 0\quad {p}_{R} >{p}_{F}\end{array}\right.$$

where ${\mathrm{L}}_{\mathrm{R}}$ is the likelihood ratio statistic for the SNP-environment interaction effect under the random effect model with overlapping data, and ${p}_{R}$ and ${p}_{F}$ are the P-values for test of the SNP-environment interaction effect applying the ROMR and OMR. The P-value for this statistic is similar to that used in Lee^17^.

When the data between studies are independent, the correlation matrix $\mathrm{C}$ becomes an identity matrix; that is to say, $\mathrm{C}=\mathrm{I}$. In this context, the method becomes RMR. However, as a result of the additional judgement process of the correlation matrix, the calculation amount of this method is increased. Therefore, the use of RMR is recommended when the data between studies are independent, while ROMR is recommended when the data is overlapping or it is not certain whether there is overlapping data.

We performed a simulation where the number of overlapping individuals were increased from 100 to 200, 300, and 400. Simulations with 500 or more overlapping individuals were not performed because there are 1000 individuals in our studies. That is, if there are 500 or more overlapping individuals between studies, none of the studies have individuals that are not applied to other studies, thus the correlation matrix cannot be guaranteed to be strictly diagonally dominant. In this case, the non-singularity of the variance matrix cannot be guaranteed; thus, this situation is not considered.

In the present study, we simultaneously evaluated the fixed effect and random effect of the SNP-environment interaction or the fixed effect and random effect of the SNP and SNP-environment interaction. When heterogeneity is high and overlapping data exists, our method provides accurate and valid power. However, our method also has some limitations. First, the calculation cost of our ROMR is much higher than that of the OMR. Second, more than one environmental variable may interact with the genetic effect being tested. Here, only one environmental variable was chosen for the interaction analysis, but other environmental variables can be entered as covariates.

## Conclusion

This study generalized the RMR proposed in our previous paper to account for overlapping data. This method was designed to test the SNP-environment interaction effect or the SNP and SNP-environment joint effects with overlapping data. To this end, a correlation matrix was introduced into our random effect model. In addition, a new method to solve the likelihood function was proposed, which allowed the solution to $\mathrm{ln}\left|V\right|$ to be obtained more easily. By simulation, we verified that our method was suitable under the conditions of the random effect model of the SNP-environment interaction with overlapping data. As a result, our ROMR obtained a higher power than OMR when the heterogeneity was high. In practice, when data from large-scale meta-analyses originate from different factors, including ethnicities, environments, phenotypes, or some other factors, heterogeneity across studies is likely to exist, our proposed ROMR can be applied.
